# Methylphenidate in Post-Stroke Rehabilitation: A Systematic Review and Meta-Analysis of Randomized Controlled Trials

**DOI:** 10.7759/cureus.97053

**Published:** 2025-11-17

**Authors:** Abinivesh Sathyanarayanan, Sunil Vummiti, Afreen Pudukayil Pudiyamaliyakkal

**Affiliations:** 1 Cardiology, Worcestershire Acute Hospitals NHS Trust, Worcester, GBR; 2 Diabetes and Endocrinology, The Royal Wolverhampton NHS Trust, Wolverhampton, GBR; 3 Medicine and Surgery, Aston University, Birmingham, GBR

**Keywords:** methylphenidate (mph), neuro rehabilitation, post-stroke mood disorder, post-stroke recovery, stroke medicine

## Abstract

Cerebrovascular disease carries significant morbidity and mortality worldwide. Medical practice has come a long way in optimizing risk factors, managing acute stroke, and preventing recurrence. There are a multitude of complications, ranging from spasticity and neuropathic pain to behavioural changes. The post-stroke period typically consists of medical management of symptoms and physical rehabilitation to recover motor function. Many patients also require occupational therapy and holistic support. Therefore, patients who have suffered a stroke require a multi-disciplinary approach to management; this is often lengthy and resource-intensive. There has been much research into interventions aimed at reducing patient morbidity and improving their quality of life. A systematic review of randomized controlled trials and a meta-analysis of results were carried out to assess the effects of methylphenidate, a dopamine and noradrenaline reuptake inhibitor, on post-stroke rehabilitation, keeping in consideration functional outcome measures and effect on mood. Limitations encountered include the quality of studies, small sample sizes, differences in treatment protocol, and varied outcome measures. There is some evidence to suggest methylphenidate may have a positive effect on mood, but there were no significant results to support its use in functional recovery. There is a need for larger trials with more sensitive and standardized outcome measures.

## Introduction and background

Stroke remains a leading cause of death and disability, affecting 100,000 people per year in the United Kingdom. This places a significant burden, approximately £3 billion annually, on the NHS, both in its acute management and in dealing with its sequelae [1]. Despite rapid advancements in its prevention and treatment with novel therapies such as Sanbexin® (edaravone and dexborneol), glibenclamide, etc. being studied, research into post-stroke rehabilitation lags behind [2,3]. 

The ongoing search for pharmacological agents to enhance neuroplasticity and motor recovery post-stroke is a key research area. Various medications and device-based therapies are under investigation. However, currently there are no guidelines on the use of these options to supplement conventional therapy.

This systematic review and meta-analysis aim to investigate the use of methylphenidate in rehabilitation after stroke, specifically with motor function. Secondarily, effects on mood and adverse effects of treatment will also be considered. A quarter of strokes occur among the working population; depending on its severity, it may ruin their careers and affect their support network. Managing disability, both physical and psychological, could play a key role in dealing with this [1].

Theory

Methylphenidate functions as a CNS stimulant by blocking reuptake of catecholamine transporters, increasing synaptic concentration of noradrenaline and dopamine, and prolonging their effects [4]. One rationale for its use in post-stroke recovery is to counteract post-stroke diaschisis, which includes functional suppression of the catecholaminergic system [5]. Clinical studies suggest that methylphenidate enhances activity in underactive neural networks after stroke, particularly in prefrontal and sensorimotor regions [6]. Subsequent cognitive benefit may be seen across domains such as inattention, working memory, and motivation, which are crucial for effective participation in post-stroke rehabilitation [7].

On a cellular level, pre-clinical studies suggest methylphenidate promotes neuroplasticity by increasing neurite outgrowth, dendritic complexity, and synaptic plasticity. These processes provide a pathway for the structural remodelling that will precede functional recovery, though the application of these findings to humans requires further investigation [8,9].

## Review

Methods

This review was conducted in accordance with the Preferred Reporting Items for Systematic Reviews and Meta-Analyses (PRISMA) guidelines [10].* *It was registered with the International Prospective Register of Systematic Reviews (PROSPERO) (registration number: CRD420251123817). The initial search was carried out on June 23, 2025, and covered a period from inception to this date.

Search Strategy

The Population/Patient, Intervention, Comparison, and Outcome (PICO) framework was used to formulate the research question and the search terms. The search included a combination of terms: ‘Methylphenidate’, ‘Cerebrovascular accident’, and ‘Rehabilitation’ or ‘mood’, and their variations. Medical Subject Headings (MeSH) terms were used when available. The PICO definitions have been summarized in Table 1.

**Table 1:** PICO framework PICO: Population/Patient, Intervention, Comparison, and Outcome; PHQ-9: Patient Health Questionnaire-9; GAD-7: Generalized Anxiety Disorder-7

Category	Items
Population	Ischemic stroke patients (≥ 18 years old)
Intervention	Methylphenidate
Comparator	Control group
Outcome	Functional recovery (Rankin scale, Barthel Index, etc.), secondary outcome - mood (PHQ-9, GAD-7, etc.), adverse effects of treatment

The search was conducted across three databases: PubMed, Scopus, and Cochrane Central Register of Controlled Trials (CENTRAL). In addition, references of relevant papers were screened to identify publications. The search strings used in the three databases can be found below.

PubMed search string: ((stroke) OR (cerebrovascular accident) OR (cerebrovascular disease)) AND (methylphenidate) AND ((rehabilitation) OR (recovery) OR (outcome) OR (neuroplasticity) OR (mood) OR (anxiety) OR (depression))

Scopus search string: TITLE-ABS-KEY("stroke" OR "cerebrovascular accident" OR "cerebrovascular disease") AND TITLE-ABS-KEY("methylphenidate") AND TITLE-ABS-KEY("rehabilitation" OR "recovery" OR "outcome" OR "neuroplasticity" OR "mood" OR "anxiety" OR "depression")

CENTRAL search string: "stroke" OR "cerebrovascular accident" OR "cerebrovascular disease" in Title Abstract Keyword AND "methylphenidate" in Title Abstract Keyword AND "recovery" OR "rehabilitation" OR "outcome" OR "neuroplasticity" OR "depression" OR "anxiety" OR "mood" in Title Abstract Keyword.

For more information on the search strategy, please view the PROSPERO record.

Eligibility Criteria

Inclusion criteria: Peer-reviewed studies were eligible for inclusion if they discussed human adults with ischemic stroke treated with methylphenidate as part of a randomized controlled trial (RCT). They must also report outcome measures that represent the functional status of patients (e.g., Rankin Scale, Barthel Index, National Institutes of Health Stroke Scale (NIHSS)) or mood (e.g., Patient Health Questionnaire-9 (PHQ-9) and Generalized Anxiety Disorder-7 (GAD-7)).

Exclusion criteria: Studies were excluded if they were other systematic reviews, abstracts, or background articles that simply discussed the use of methylphenidate post stroke. This was classed as ‘wrong publication’; however, their references were screened to find relevant RCTs. Non-English papers were not included and reported in the PRISMA flowchart as 'foreign language'. Papers that did not have full-text available even after attempting to contact authors were also excluded as 'reports not received'.

Study Selection

Search results were imported to Rayyan [11]. It allowed automated removal of duplicates (cross-checked by authors) and it offered blinds for the reviewers. Results underwent title and abstract screening in the first instance, and then full-text screening. Both of these steps were carried out by two independent blinded authors (AS, SV).

Exclusion reasons were used with a syntax to make the screening reproducible. Papers were first checked to see if the subjects were human adults with ischemic stroke; if not, they were marked as ‘wrong population’. If they did meet the population criteria, they were then evaluated for mention of methylphenidate; if not, marked as 'wrong intervention'. Finally, if they did not discuss outcome measures for function or mood, they were marked as 'wrong outcome'. If a paper met all three of these criteria but was not an RCT or discussing one, it was classed as 'wrong publication type'. During screening, many abstracts, background articles, and observational studies fell under this criterion. Any conflicts were resolved through discussion with a third reviewer (AP).

**Figure 1:**
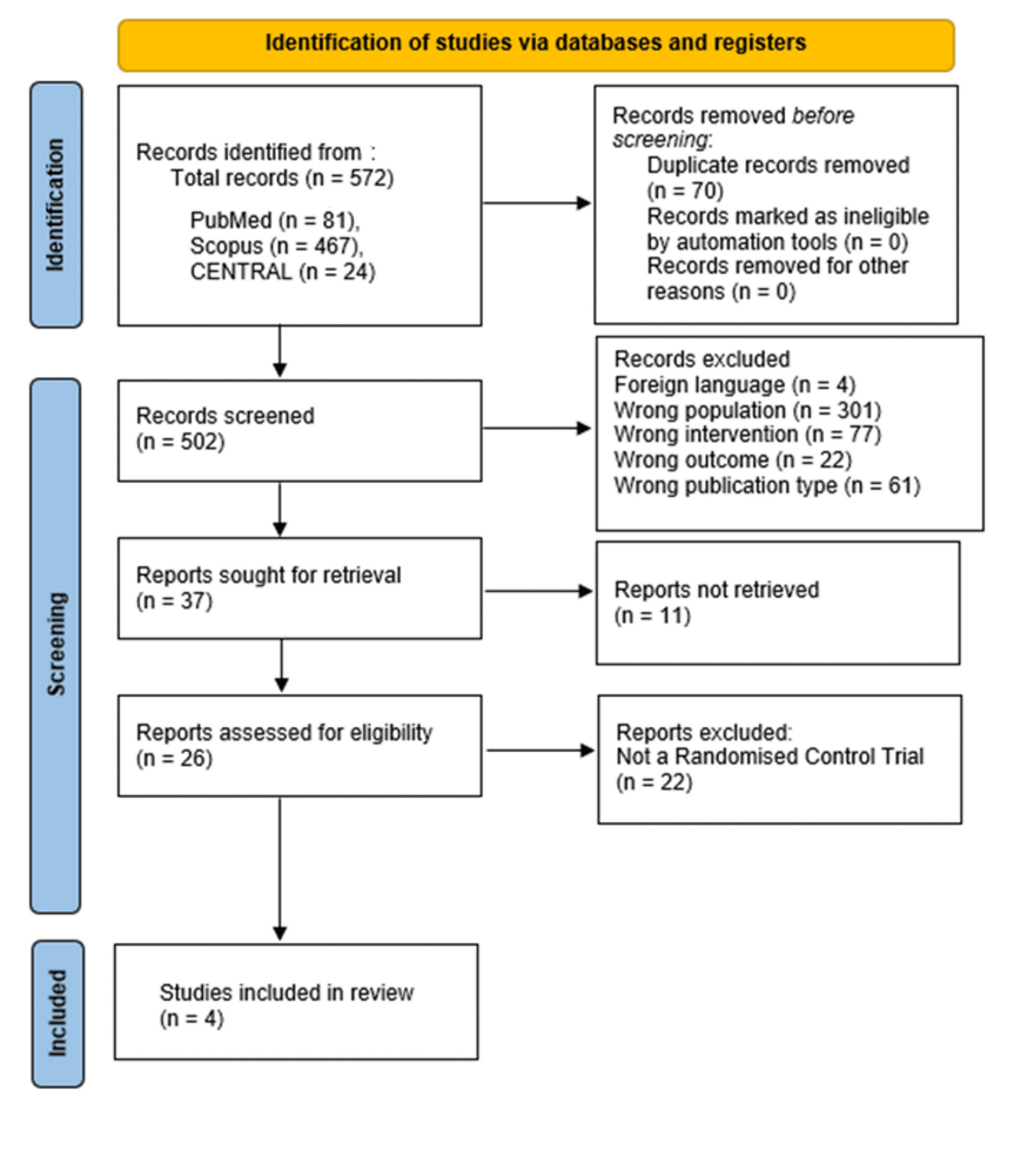
PRISMA flowchart PRISMA: Preferred Reporting Items for Systematic Reviews and Meta-Analyses

In total, 26 papers were selected for full-text screening and assessed for eligibility. Most of these were systematic reviews, and their references also underwent screening. Four RCTs met the inclusion criteria and are discussed in this review [6,12-14]. The PRISMA flowchart for the search and study selection can be found in Figure 1.

Data Extraction

Data were extracted from the included trials and transcribed into a pre-defined table. This was done by all the reviewers together (AS, SV, AP). Information on demographics (age, sex) and their distribution, pre- and post-treatment scores on assessment of functional status and mood, including their mean and standard deviations, are summarized in Table 2. These values were used for further meta-analysis.

**Table 2:** Data extraction NIHSS: National Institutes of Health Stroke Scale

Author (Year)	Intervention Age, mean±SD	Control Age, mean±SD	Sex, n	Scores	Intervention Number (Ni)	Intervention Mean (Mi)	Intervention SD (Sdi)	Control Number (Nc)	Control Mean (Mc)	Control SD (Sdc)
Grade et al., 1998 [12]	69.8±3.66	72.64±3.49	Intervention: M=6, F=6; Control: M=7, F=4	Hamilton Depression Rating Scale (HAM-D)	10	14.316	1.522	11	19.455	1.488
				Zung Self-Rating Depression Scale	10	32.949	2.123	11	38.97	2.024
				Fugl-Meyer Scale (FMS)	10	55.259	7.073	11	37.777	4.994
				modified Functional Independence Measure (M-FIM)	10	116.46	3.569	11	104.945	3.401
Delbari et al., 2011 [13]	64.05±10.8	65.3±9.6	Intervention: M=9, F=10; Control: M=14, F=6	Geriatric Depression Scale						
				At 15 days	19	5.1	1.7	20	5.7	2.1
				At 90 days	19	4.5	1.9	20	5.7	2.2
				At 180 days	19	4.3	2	20	5.7	2.1
Lokk et al., 2011 [14]	64.05±10.8	65.3±9.6	Intervention: M=9, F=10; Control: M=14, F=6	Fugl-Meyer Score (FMS)	19	57	35.5	20	53.4	34.4
				Barthel index (BI)	19	71.58	16	20	70.5	14.4
				NIHSS	19	2.9	2.6	20	4	3.6
Tardy et al., 2006 [6]	63±11.40	58±8.04	Intervention: M=4; Control: M=4	NIHSS	4	0.5	0.577	4	0.5	0.577

Additional information on the stroke event (classification, laterality, duration), the trial design, and the treatment itself (dosing regimen) was sought, though the trials do not report these consistently. Adverse effects of treatment were also looked at carefully.

Assessment of Bias

The Cochrane Risk of Bias 2 (RoB2) tool was used to assess the quality of the included trials [15]. The study design and RoB results can be found in Table 3. More thorough details on the RoB2 domains can be found in Table 4.

**Table 3:** RoB2 tool domains D1 - D5 L: low risk; C: some concerns; H: high risk; NIHSS: National Institutes of Health Stroke Scale; MPH: methylphenidate

Study	Study design	Intervention	Comparator	Outcome	D1	D2	D3	D4	D5	Overall
Grade et al., 1998 [12]	Prospective, randomized, double-blind, placebo-controlled study	Increasing dose of MPH (5 mg capsules). Day 1 - one capsule at 08:00, Day 2 - two capsules at 08:00 and 12:00. Then the dose was increased by one capsule every 3 days. Maximum dose - 30 mg/day, three capsules BD. Maximum duration of treatment is 3 weeks. Patients also underwent physical therapy.	Control	Fugl-Meyer Scale, Hamilton Depression Rating Scale, Zung Self-Rating Depression Scale	L	L	L	C	H	H
Delbari et al.,2011 [13]	Prospective, randomized, double-blind, placebo-controlled trial	MPH at 20 mg per day for 5 days a week for 3 weeks. This was coupled with training. The outcomes are reported at 15, 90, and 180 days.	Control	Geriatric Depression Scale	L	L	L	C	L	C
Lokk et al., 2011 [14]	Prospective, randomized, double-blind, placebo-controlled trial	MPH at 20mg per day for 5 days a week for 3 weeks. It was administered 60 minutes before training sessions. The outcomes are reported at 3 and 6 months. We will include the results at 3 months.	Control	NIHSS, Fugl-Meyer	L	L	L	C	C	C
Tardy et al., 2006 [6]	Prospective, randomized, double-blind, placebo-controlled trial	MPH was given as a single dose of 20 mg, and this was coupled with training. Patients were assessed 17.5 days (median) after stroke onset.	Control	NIHSS	L	L	L	C	C	C

**Table 4:** RoB2 tool - domains and signalling questions Y/PY/NI: Yes, Probably Yes, and No Information

Domain	Signalling question		
Bias arising from the randomization process	1.1 Was the allocation sequence random?		
	1.2 Was the allocation sequence concealed until participants were enrolled and assigned to interventions?		
	1.3 Did baseline differences between intervention groups suggest a problem with the randomization process?		
	Risk of bias judgement		
Bias due to deviations from intended interventions	2.1.Were participants aware of their assigned intervention during the trial?		
	2.2.Were carers and people delivering the interventions aware of participants' assigned intervention during the trial?		
	2.3. If Y/PY/NI to 2.1 or 2.2: Were there deviations from the intended intervention that arose because of the experimental context?		
	2.4 If Y/PY to 2.3: Were these deviations likely to have affected the outcome?		
	2.5. If Y/PY/NI to 2.4: Were these deviations from intended intervention balanced between groups?		
	2.6 Was an appropriate analysis used to estimate the effect of assignment to intervention?		
	2.7 If N/PN/NI to 2.6: Was there potential for a substantial impact (on the result) of the failure to analyse participants in the group to which they were randomized?		
	Risk of bias judgement		
Bias due to missing outcome data	3.1 Were data for this outcome available for all, or nearly all, participants randomized?		
	3.2 If N/PN/NI to 3.1: Is there evidence that result was not biased by missing outcome data?		
	3.3 If N/PN to 3.2: Could missingness in the outcome depend on its true value?		
	3.4 If Y/PY/NI to 3.3: Is it likely that missingness in the outcome depended on its true value?		
	Risk of bias judgement		
Bias in measurement of the outcome	4.1 Was the method of measuring the outcome inappropriate?		
	4.2 Could measurement or ascertainment of the outcome have differed between intervention groups?		
	4.3 Were outcome assessors aware of the intervention received by study participants?		
	4.4 If Y/PY/NI to 4.3: Could assessment of the outcome have been influenced by knowledge of intervention received?		
	4.5 If Y/PY/NI to 4.4: Is it likely that assessment of the outcome was influenced by knowledge of intervention received?		
	Risk of bias judgement		
Bias in selection of the reported result	5.1 Were the data that produced this result analysed in accordance with a pre-specified analysis plan that was finalized before unblinded outcome data were available for analysis?		
	5.2 ... multiple eligible outcome measurements (e.g. scales, definitions, time points) within the outcome domain?		
	5.3 ... multiple eligible analyses of the data?		
	Risk of bias judgement		
Overall bias	Risk of bias judgement		

Analysis

Meta-analysis was performed with a random effects model on R software (R Foundation for Statistical Computing, Vienna, Austria, https://www.R-project.org/). Heterogeneity was assessed with the tau-squared (𝜏^2^)statistic (Restricted Maximum Likelihood procedure), and prediction intervals were also calculated. Cochran’s Q test and I^2^ statistic are also reported. Mean differences (MD) are the effect sizes of choice. Results are used to generate forest plots, which were used alongside the I^2^ statistic to determine if outliers exist. No subgroup analysis was carried out.

Results

Looking at data extracted from the included studies, there were significant differences in recruitment, dosing regimen, time to treatment, and its duration, supplementary physiotherapy, and scales used in assessment. This significantly limited the results of the meta-analysis. There was also concern regarding the quality of the included studies as shown by the RoB2 Tool.

This review reports on motor outcomes with MDs in NIHSS [12,14] and Fugl-Meyer Scale [6,14]. We would like to call attention to using subjects from Lokk et al. [14] twice; however, they are used independently in two different calculations for both the scores. Delbari et al.'s trial, which assesses the effect on mood with the Geriatric Depression Scale (GDS), is also discussed [13]. It appears that both these studies were done on the same population. Grade et al. also assessed the Hamilton Depression Rating Scale score (HAM-D) and the Zung Self-Rating Depression Scale (ZDS) [12].

Motor Outcome

**Figure 2:**
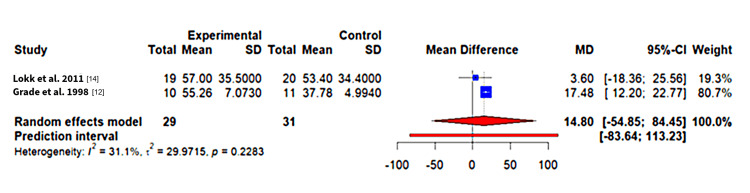
Fugl-Meyer scores (higher scores favours intervention) Reference: [12,14]

With regards to the Fugl-Meyer scores, there is an MD of 14.8 (n = 60, p-value = 0.2258, 95%CI, -54.85 to 84.45). This is positive and so supports the intervention; however, it is not a statistically significant result [16]. It is less meaningful to try and interpret heterogeneity with two studies, but we still report it. There is mild-moderate heterogeneity with 𝜏^2^ = 29.97, I^2^ = 31.1% and Q test = 1.45 (p-value = 0.2283, d.f. = 1). The 95% prediction interval was calculated to be -83.64 to 113.23; as this includes zero, we cannot be sure about the effects of future studies. The forest plot for this analysis can be found in Figure 2. It shows that the study by Grade et al. 1998 has a marked positive effect, being mindful of the high risk of bias as flagged by the RoB2 tool; however, the overall estimate crosses the line of no effect [12].

**Figure 3:**
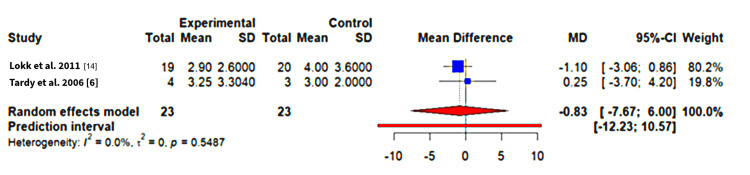
NIHSS (lower scores favours intervention) NIHSS: National Institutes of Health Stroke Scale References: [6,14]

An MD of - 0.83 was seen in the NIHSS scores (n = 46, p-value = 0.54, 95%CI, - 7.67 to + 6.00); this favors the intervention. This result is again not statistically significant. There is no significant heterogeneity seen between studies when using the restricted maximum likelihood procedure. This is likely due to the small sample size, which does not allow 𝜏^2^ calculation. The forest plot for this analysis is available in Figure 3. It shows that while the individual study effects are in the expected direction, they cross the line of no-effect [6,14].

Mood

The study by Delbari et al. shows an MD of - 0.98 in GDS score at 15 days (n = 39, p-value = 0.33, 95%CI, - 1.83 to + 0.63) and at 90 days, there was an MD of -1.20 (p-value = 0.075, 95%CI, -2.53 to + 0.13). However, at the last follow-up at 180 days, the MD was -1.40 (p-value = 0.033, 95%CI, - 2.73 to - 0.06), which is significant [13]. Grade et al. used two tools to determine improvement in mood. With the HAM-D, an MD of -5.13 (p-value <0.00001, 95%CI, -6.51 to -3.76) was observed. The ZDS identified an MD of -6.02 (p-value <0.00001, 95%CI, -7.91 to -4.12) [12].

From the meta-analysis results, no outliers were identified, and no subgroup analysis was carried out. The results described above do not provide enough evidence to support the use of methylphenidate to improve motor recovery. The side effect profile of the medication, however, makes it an attractive option for managing low mood post-stroke.

Adverse Effects

Grade et al. measured adverse drug reactions in their patients using a checklist and reported no statistically significant difference in the number of side effects between the participants on methylphenidate and those on placebo. They do not report the individual incidences [12]. Tardy et al. screened for cardiovascular (risk of raised blood pressure, tachycardia), neurological (nervousness, insomnia, and rare dyskinesia), and gastrointestinal side effects. There was no incidence of these in their sample [6]. Delbari et al. [13] and Lokk et al. [14] did not formally assess side effects.

Discussion

The four studies included in this review were found to have significant differences between them. This includes how patients were selected and the time to treatment. Tardy et al. only included patients with pure motor hemiparesis and had a time to treatment of 17.5 days (median) [6], Lokk et al. [14] and Delbari et al. [13] included those with chronic limb (arm or leg) paresis with a mean of 66.26 days (SD = 40.7 days) post stroke, and finally Grade et al. did not place any restrictions as long as patients were able to complete test instruments; patients received methylphenidate at a mean of 17.9 days (SD = 3.74) [12]. The different dosing regimes used are summarized in Table 3. These patients are also exposed to varying degrees of physiotherapy, influenced by local policies and engagement. This contributes to inter-study heterogeneity and the generalizability of results.

There were concerns raised by the RoB2 tool, which indicated that the RCT by Grade et al. [12] was at a high risk of bias, while the others showed some concerns. This affects the quality of this meta-analysis and the overall estimate of true effect sizes.

Expected positive direction of effects was seen across all studies, with the exception of Tardy et al. [6]. This might be explained by the small sample size and the use of the relatively insensitive NIHSS. The outcome measures used to assess motor recovery need to be sensitive enough to detect subtle improvements. The Fugl-Meyer scale might be more suitable in this regard as it scores specific upper and lower limb movements; furthermore, it was also found to have high inter-rater reliability [16]. Results from Grade et al. showed a large and positive change in the Fugl-Meyer Scale (high risk of bias) [12]. The overall results, however, were not significant to support methylphenidate use to improve motor function as assessed with the NIHSS and Fugl-Meyer Scale [6,12,14].

Studies have shown that the presence of depression in stroke patients is associated with poorer functional outcomes. This might be explained by difficulties in engaging with rehabilitation [9]. Grade et al. found significant improvements in both the HAM-D as well as the ZDS [12]. In the study done by Delbari et al., there is an improvement in mood at 180 days (as assessed with the GDS) [13]. This result might indicate that patients will need to receive a prolonged course of treatment to observe beneficial effects. This is problematic given how the use of stimulants is known to increase heart rate and blood pressure. In the typical stroke patient with pre-existing comorbidities, this could be a contraindication [17]. The studies by Grade et al. [12] and Tardy et al. [6] found no adverse drug reactions; these followed patients over a relatively short period of time (three weeks), and the latter only used a single dose of methylphenidate.

Strokes are the leading cause of long-term disability worldwide [18]. The evidence gathered in this systematic review, while positive, does not support the use of methylphenidate in routine practice. Clinical trials with larger samples, standardized recruitment and treatment regimens, and long-term follow-up for adverse effects are needed.

## Conclusions

This systematic review found a small number of RCTs investigating methylphenidate use in patients for post-stroke rehabilitation. These had significant inter-study differences, which limited the meta-analysis of their results. While the effect sizes improved, keeping with the hypothesis, this was not significant for motor outcomes. There is some evidence supporting improvement in mood. However, we are currently unable to draw conclusions on the effects of treatment. This review highlights the need for further research in this area, specifically clinical trials. Given the morbidity of strokes and the apparent side-effect profile of the medication, it is a viable option to consider.
